# Results of 2‐Year Ring Testing of a Semifield Study Design to Investigate Potential Impacts of Plant Protection Products on the Solitary Bees *Osmia Bicornis* and *Osmia Cornuta* and a Proposal for a Suitable Test Design

**DOI:** 10.1002/etc.4874

**Published:** 2020-11-16

**Authors:** Lea Franke, Charlotte Elston, Tobias Jütte, Olaf Klein, Silvio Knäbe, Johannes Lückmann, Ivo Roessink, Markus Persigehl, Magdaléna Cornement, Nina Exeler, Hervé Giffard, Bettina Hodapp, Stefan Kimmel, Britta Kullmann, Christof Schneider, Alexander Schnurr

**Affiliations:** ^1^ Eurofins Agroscience Services Ecotox, Niefern‐Öschelbronn Germany; ^2^ Syngenta, Jealott's Hill International Research Centre Bracknell Berkshire United Kingdom; ^3^ Institute for Bee Protection, Julius Kühn‐Institut Federal Research Centre for Cultivated Plants, Messeweg Braunschweig Germany; ^4^ Rifcon Hirschberg Germany; ^5^ Wageningen Environmental Research Wageningen The Netherlands; ^6^ tier3 solutions Leverkusen Germany; ^7^ Innovative Environmental Services Witterswil Switzerland; ^8^ Bayer CropScience Monheim Germany; ^9^ Testapi, Gennes, Gennes‐Val‐de‐Loire France; ^10^ BASF Limburgerhof Germany; ^11^ BioChem agrar, Gerichshain Germany

**Keywords:** Ecotoxicology, Non‐*Apis*, *Osmia*, Semifield test design, Pesticides, Risk assessment

## Abstract

There are various differences in size, behavior, and life history traits of non‐*Apis* bee species compared with honey bees (*Apis mellifera*; Linnaeus, 1758). Currently, the risk assessment for bees in the international and national process of authorizing plant protection products has been based on honey bee data as a surrogate organism for non‐*Apis* bees. To evaluate the feasibility of a semifield tunnel test for *Osmia bicornis* (Linnaeus, 1758) and *Osmia cornuta* (Latreille, 1805), a protocol was developed by the non‐*Apis* working group of the International Commission for Plant‐Pollinator Relationships, consisting of experts from authorities, academia, and industry. A total of 25 studies were performed over a 2‐yr period testing a replicated control against a replicated positive control using either a dimethoate or diflubenzuron treatment. Studies were regarded to be valid, if ≥30% of released females were found to occupy the nesting units in the night/morning before the application (establishment). Thirteen studies were regarded to be valid and were analyzed further. Parameters analyzed were nest occupation, flight activity, cell production (total and per female), cocoon production (total and per female), emergence success, sex ratio, and mean weight of females and males. Dimethoate was a reliable positive control at the tested rate of 75 g a.i./ha, once >30% females had established, displaying acute effects such as reduction in flight activity, increase in adult mortality (shown by nest occupation), and reproduction ability of the females (total cell and cocoon production). On the other hand, no effects on larval and pupal development were observed. The growth regulator diflubenzuron had statistically significant effects on brood development, causing mortality of eggs and larvae at a rate of approximately 200 g a.i./ha, whereas fenoxycarb did not cause any significant effects at the tested rates of 300 and 600 g a.i./ha. In conclusion, the ring‐test protocol proved to be adequate once the study comprised a well‐established population of female *Osmia* bees, and the results improved in the second year as the laboratories increased their experience with the test organism. It is noted that the success of a study strongly depends on the experience of the experimenter, the crop quality, the quality of the cocoons, and the weather conditions. Based on these finding, recommendations for a semifield study design with *Osmia* spp. are proposed. *Environ Toxicol Chem* 2021;40:236–250. © 2020 The Authors. *Environmental Toxicology and Chemistry* published by Wiley Periodicals LLC on behalf of SETAC.

## Graphical abstract

For ecotoxicological testing of pesticides, a protocol was developed and ring‐tested by the non‐*Apis* working group of the International Commission for Plant‐Pollinator Relationships to conduct semifield tunnel tests with the solitary bee species *Osmia bicornis* (Linnaeus, 1758) and *Osmia cornuta* (Latreille, 1805).

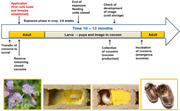

## INTRODUCTION

Recent reports of the loss of insect biodiversity have received significant public attention (Potts et al. 2010; Goulson et al. 2015). Of all insect species, bees (Hymenoptera: Apiformes) are of particular interest, as they are so‐called keystone species, providing an essential ecosystem service: pollination (Rathcke and Jules 1993; Klein et al. 2006; Benedek et al. 2007; Garibaldi et al. 2013). The loss of these keystone species could have many negative ecological and economic consequences (Pimentel et al. 1997; Chagnon et al. 2015). Important factors driving these declines are habitat loss due to the intensification of agriculture, the expansion of urban areas, climate change, and the introduction of invasive plant and insect species (Potts et al. 2010; Goulson et al. 2015; Crenna et al. 2016; Hladik et al. 2016). Associated with the intensification of agriculture are both 1) the loss of nesting sites and forage in agricultural landscapes, and 2) the use of plant protection products, which can pose a risk to bees. The 2 main exposure routes of bees to plant protection products are contact exposure, for example, through direct overspray during an application or through contaminated nesting material, or oral exposure through residues in pollen and nectar (European Food Safety Authority 2013; Sgolastra et al. 2018).

In the European Union all plant protection products have to be registered and approved under Regulation (EC) 1107/2009 (European Commission 2009) before they can be placed on the market. At present one species, the European honey bee (Hymenoptera: Apidae. *Apis mellifera* L.), is used as a surrogate species to assess the risk of plant protection products to bees. There remains discussion about whether this approach is protective of non‐*Apis* bees (Heard et al. 2017; Lewis and Tzilivakis 2019; Thompson and Pamminger 2019). Non‐*Apis* bees comprise a wide range of body sizes as well as biological and life history traits, which may result in differences in sensitivity and exposure routes in comparison with honey bees (Biddinger et al. 2013; Arena and Sgolastra 2014; Thompson 2015; Uhl et al. 2016; Gradish et al. 2018; Sgolastra et al. 2018; Bireley et al. 2019; Boyle et al. 2019). For instance, in contrast to honey bees, approximately 65% of non‐*Apis* bee species build their nests in the soil or use plant components such as leaves and resin as nesting material. Both exposure routes are not well understood so far and thus are not (yet) considered in the current risk assessment. Moreover, because every single female represents a reproducing unit in solitary bees, the death of every nesting female results automatically in the loss of her progeny. This is in contrast to social bees, which are able to compensate for the loss of worker bees to a certain degree (Sgolastra et al. 2018). Therefore effects on solitary bee individuals could have a very different impact on their population compared with social species.

In 2013 the European Food Safety Authority (EFSA) published a Guidance Document on bees, which recommended considering not only honey bees, but also bumble bees and solitary bees in the plant protection product risk assessment. For solitary bees, the EFSA advised the use of the closely related mason bee species *Osmia cornuta* (Latreille, 1805) and *Osmia bicornis* (Linnaeus, 1758; Hymenoptera: Megachilidae). At the time of the publication of the EFSA Bee Guidance Document, no suitable methods or guidelines were available to generate reliable data for the risk assessment of plant protection products on non‐*Apis* species, for either lower tier laboratory studies or under more realistic conditions in higher tier semifield or field studies.

To address these knowledge gaps, the International Commission for Plant‐Pollinator Relationships (ICP‐PR) established a non‐*Apis* working group in 2014. It consists of experts from authorities, academia, and industry and aimed to develop and establish robust and reproducible test methods for solitary bee (*Osmia* sp.) testing under laboratory, semifield, and field conditions.

In terms of higher tier studies with solitary nesting bee species (i.e., semifield and field tests) reports of using *Osmia lignaria* (Say, 1837), *O. bicornis, Megachile rotundata* (Fabricius, 1784; Hymenoptera: Megachilidae), and *Nomia melanderi* (Cockerell, 1906; Hymenoptera: Halictidae) are available in the literature (Torchio 1983; Mayer et al. 1998; Alston et al. 2007; Abbott et al. 2008; Ladurner et al. 2008; Hodgson et al. 2011; Artz and Pitts‐Singer 2015; Rundlöf et al. 2015; Peters et al. 2016; Ruddle et al. 2018). All 4 species are bred and managed commercially for pollination services (Sgolastra et al. 2018). Of these 4 species *M. rotundata* and *O. bicornis* are native to Europe, of which only *Osmia* is commercially available in Europe. As well as the differences in bee species, the experimental methods used in the published studies were heterogeneous as well. As a result, no overarching methodology could be derived from the literature. Both *O. bicorni*s and *O. cornuta* display a pronounced polylectic feeding behavior. It has been observed that these species, when free‐flying in field studies, will collect pollen from a range of sources other than the treated crop, resulting in low exposure of adults and larvae to a test substance in the treated crop (Peters et al. 2016; Ruddle et al. 2017). To ensure sufficient exposure in the trials, the working group decided to focus on a tunnel set‐up with bees being enclosed on the treated crop to produce more comparable and standardizable results.

After several pretests in 2014 and 2015, a first test protocol for semifield testing of solitary bee species (i.e., *O. bicornis* and *O. cornuta*) was developed in 2016 by the non‐*Apis* working group and was then field‐tested in 2016 and 2017 based on the experience obtained (International Commission for Plant‐Pollinator Relationships 2016, 2017). The protocol is based on the European and Mediterranean Plant Protection Organization (2010) guideline no. 170(4) and general Society of Environmental Toxicology and Chemistry/European Standard Characteristics of Non‐target Arthropod Regulatory Testing recommendations (Barrett et al. 1994). It generally resembles semifield studies conducted with honey bee colonies (Organisation for Economic Co‐operation and Development 2007), but was adjusted to the needs and requirements of solitary cavity‐nesting bee species. The final protocol can be found in the Supplemental Data, Table SI‐1.

The following objectives of the ring test were defined: 1) to establish a standard experimental design for semifield testing of mason bees (*O. bicornis* and *O. cornuta*); 2) to establish suitable reference substances (positive controls); and 3) to establish reliable and reproducible study parameters

## MATERIALS AND METHODS

Ring‐test studies were conducted in 2016 and 2017 by 9 laboratories from Germany, Switzerland, and France, which performed a total of 25 studies.

### Test organisms

**Figure 1:**
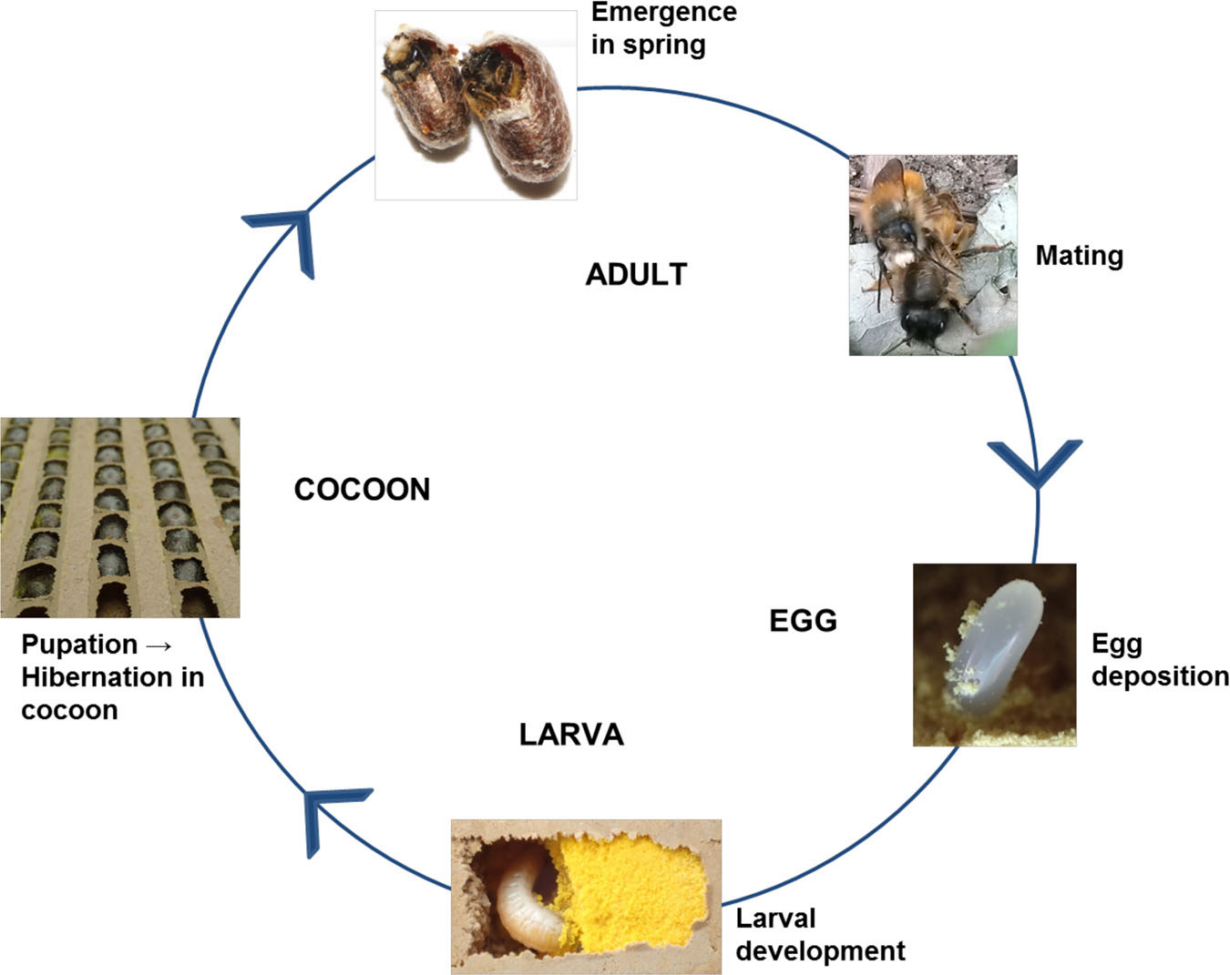
Life cycle of *Osmia bicornis*. Adults are emerging from cocoons in spring and females start the deposition of eggs after mating. Larvae are hatching from eggs and start feeding on the pollen provision in the brood cell. Before hibernation the larvae spin a cocoon, in which they overwinter and complete their maturation. In the next spring, the adult bees emerge from cocoons.

The red mason bee *O. bicornis* was selected as a test organism. However, some laboratories conducted additional studies with *O. cornuta*, to test whether the study design would also work with other *Osmia* species. Both species were selected because they are polylectic species native to Europe (Peters 1978) and are readily available from commercial suppliers. They naturally nest between March and May (*O. cornuta*) or March and June (*O. bicornis*). In spring the bees start to emerge from cocoons, in which they overwintered as prepupae (see Figure 1). Males emerge a few days before the females (protandry). After mating several times the females start to build nests in pre‐existing cavities using moist soil as nesting material. Each female builds up to 30 brood cells consisting of a provision of pollen mixed with nectar and a single egg (Amiet and Krebs 2012; Scheuchl and Willner 2016). Only the females take care of the brood, meaning that reproductive success mainly depends on the vitality of the females.

Bees of both test species were obtained as cocoons from local breeders and kept under cooled (2 ± 2 °C) and humid (60–80%) conditions until the start of the test. Before the actual start of the study, the cocoons were incubated at 22 ± 2 °C to synchronize the emergence of the bees with the onset of the flowering of the selected crop. At least 30 females were released per tunnel either as cocoons or as adults (the maximum density was 1.2 nesting females/m²). Based on experiences from 2016, more males than females were released to ensure successful mating of all females (between 1:1.3 and 1:2.0 females:males were released).

### Test design

The semifield trials consisted of at least 2 treatment groups: one control (C) and one or more test item groups (T). The trials were conducted with 4 replicates/treatment group in 2016 and 6 replicates in 2017. The number of replicates was increased in an attempt to reduce the variability in the data. Each replicate consisted of a semifield tunnel containing either *Brassica napus* or *Phacelia tanacetifolia* as crop. The tunnel size ranged from 33 to 100 m² and the number of released females was tailored to a maximum density of 1.2 nesting females/m².

Each tunnel was equipped with one nesting unit (Supplemental Data, Figure SI‐1). These were composed of an outer chassis with a rainproof roof containing medium‐density fiberboard or plastic trays, which offered on average nesting space of 1.1 to 3.6 cavities (tubes)/released female (Supplemental Data, Figure SI‐2). In turn, each tray was covered by a transparent plastic sheet to allow the marking of constructed cells throughout the test period. The nesting units were placed well above the ground to avoid humid conditions in the cavities. The entrance of the unit was oriented southeast to capture the sun, with the aim of enhancing the activity of the bees early in the morning. Because *Osmia* females need muddy soil for the construction of the cell partitions and nest plugs, either a hole was dug next to each nesting unit with water being added regularly, or a plastic tray containing wet soil was placed close to the unit.

The incubated cocoons were placed in the nesting units just before flowering of the crop (~BBCH 59) or at the beginning of flowering of the crop (~BBCH 61). Empty cocoons of emerged bees were regularly counted to document the emergence process, after which they were removed from the trays. Right before the test item was applied, the remaining cocoons that had not emerged were taken out of the nesting units to avoid the release of bees after application and also to avoid release of parasitoids, which emerge slightly later than the bees.

In the valid studies, the test item application was conducted as soon as at least 30% of released females had established at the nesting units and started to lay the first eggs. All eggs laid before the application were excluded from further analysis, because exposure to the test item via the collected pollen and nectar had not occurred. Only after application of the test substance were the adult bees and their brood exposed to possible residues of the test item within nectar and pollen of the crop. The exposure phase lasted approximately 2 to 5 wk, depending on the crop used. At the end of flowering, the nesting units were covered with a fine gauze mesh to stop the nesting activity and to prevent parasites and predators from entering the cavities. After the exposure period in the tunnels, the development of the progeny was followed until the following spring. To allow undisturbed development of larvae, the covered nesting units were left at the field sites. If nesting units had to be removed, they were carefully transferred out of the tunnels and stored in a protected place (dry and at ambient temperatures) until cocoon formation was completed in autumn. All produced cocoons were collected from the nesting units, cleaned, and stored at 2 ± 2 °C at a mean relative air humidity of 60 to 80% for at least 3 mo (hibernation period). After this period all cocoons or a subsample of cocoons (when cocoon numbers were high a subsample of at least 80 cocoons/nesting unit was taken) were incubated to assess the emergence success of the progeny.

### Test items

The organophosphate insecticide dimethoate was chosen as the test item; it is known to be toxic to adult honey bees and is used as a reference standard in ecotoxicological studies with honey bees (European and Mediterranean Plant Protection Organization 2010; Commission des Essais Biologiques 2011). In addition, some laboratories tested the insect growth regulators diflubenzuron and fenoxycarb to establish whether they were suitable reference standards with respect to brood effects. All test items were applied during flowering and daily bee flight as a spray application following Good Agricultural Practice. In 15 of the studies, dimethoate was applied at a rate of 75 g a.i./ha. In 2 studies, dimethoate was applied at a lower rate of 25 g a.i./ha. Diflubenzuron (200 and 216 g a.i./ha) was applied in 6 of the studies, and fenoxycarb (300 and 600 g a.i./ha) was applied in 2 of the studies (Table 1). Note that the outcome of one study with fenoxycarb has already been published by Lückmann et al. (2018).

**Table 1:** Details on the participating laboratories in the ring test with the test items dimethoate, diflubenzuron, and fenoxycarb and the bee species *Osmia bicornis* and *Osmia cornuta*

	Laboratory	Test species	Test item (active ingredient)	Test item rate (g active ingredient/ha)	Data gaps	Quality criteria^a^
2016	1^b^	*O. cornuta*	Dimethoate	25	No	Fulfilled
			Diflubenzuron	216	Flight activity	Fulfilled
		*O. bicornis*	Dimethoate	25	Flight activity	Low establishment
			Diflubenzuron	216	No	Low establishment
	2	*O. bicornis*	Dimethoate	75	No	Low establishment
	3	*O. bicornis*	Dimethoate	75	No	Fulfilled
			Fenoxycarb	300	No	Fulfilled
	4	*O. bicornis*	Dimethoate	75	Flight and nesting activity	Establishment sign. different before application
	5	*O. bicornis*	Fenoxycarb	600	No	Fulfilled
	6	*O. bicornis*	Dimethoate	75	No	Fulfilled
	7	*O. bicornis*	Dimethoate	75	Cocoon production	Fulfilled
	8	*O. bicornis*	Dimethoate	75	No	Low establishment, no exposure (no flight at application)
	9	*O. bicornis*	Dimethoate	75	No	Low establishment^c^
			Diflubenzuron	216	No	Low establishment
2017	1^b^	*O. cornuta*	Dimethoate	75	No	Low establishment
			Diflubenzuron	216	Flight activity	Low establishment
		*O. bicornis*	Dimethoate	75	No	Fulfilled
			Diflubenzuron	216	No	Fulfilled
	2	*O. bicornis*	Dimethoate	75	No	Low establishment
	4	*O. bicornis*	Dimethoate	75	No	Fulfilled
			Diflubenzuron	200	No	Fulfilled^d^
	6	*O. bicornis*	Dimethoate	75	No	Fulfilled
	7	*O. bicornis*	Dimethoate	75	No	Low establishment
	8	*O. bicornis*	Dimethoate	75	No	Fulfilled
	9	*O. bicornis*	Dimethoate	75	No	Low establishment

^a^ Quality criterion: ≥30% of released females occupying the nesting units in the night/morning before the application (establishment).

^b^ Release of bees as adults.

^c^ Problems with emergence (mean emergence rate of bees from cocoons/treatment group <40%).

^d^ Only 2 replicates in the diflubenzuron treatment group.

### Assessments/parameters

A number of different assessments were performed to investigate lethal and sublethal effects on adult *O. bicornis* and *O. cornuta* and their brood:

#### Nest occupation (nesting activity)

This was assessed by counting the number of females occupying the cavities inside the nesting units after the end of bee flight or very early in the morning before bee flight. In this way the establishment of females before the application was monitored. After application the nest occupation was assessed at regular intervals (e.g., every 2nd or 3rd day) as an indirect measure of mortality until the end of the exposure phase in the tunnels.

#### Flight activity

This was noted shortly before the application to guarantee a sufficient exposure and after the application to assess behavioral and lethal effects. Therefore the number of females entering the nesting cavities in a defined time interval was counted, for example, 3 min.

#### Cell production/reproductive performance (fecundity)

This was assessed by counting the number of cells built in the nesting cavities after application. This was done either by photo documentation and/or marking on a transparent sheet. A cell is defined as an egg placed on a food provision (mass of pollen and nectar) and a mud wall as sealing. All cells built before the application, that is, complete cells and cells under construction, were excluded from further analysis, because these larvae were not exposed to pesticide residues in the food provisions.

The total number of produced cells in the test item treatment was compared with the control to determine whether the test item had an impact on the offspring number (cell production/nesting unit). The reproductive performance (fecundity) of female bees was calculated as cell production/nesting female (for the definition of females, see *Data preparation*).

#### Cocoon production

The development of the eggs was monitored until cocoon formation, and the number of cocoons was counted in autumn. In addition, the immature mortality was calculated for the studies conducted with insect growth regulators (diflubenzuron and fenoxycarb): immature mortality = % of dead eggs and larvae (calculated as difference of cocoon and cell production in % of total cell production/nesting unit).

#### Offspring production

In the following spring, after the hibernation period, the emergence success of male and female bees from overwintered cocoons was assessed. For this purpose the cocoons were incubated at 22 ± 2 °C, and the number of emerged bees was determined. All emerged bees were weighed (either individually or grouped by sex/replicate and emergence day), and the sex was determined to assess potential effects on offspring weight and the sex ratio.

### Quality criteria

For the purpose of the ring test the main quality criterion for a study was if ≥30% of released females were found to occupy the nesting units in the night/morning before the application (establishment). Note that the successful establishment of female bees at the nesting units depends on several factors, such as the material of the nesting unit and the type of release. After the experiences in 2016, recommendations were given to optimize establishment. This will be explained in the *Discussion* section. In addition, a statistically significant effect of the toxic reference (in the context of the ring tests, the test item) should be observed.

### Data preparation

To compare data between the different laboratories, some parameters were normalized as follows.

#### Nest occupation (nesting activity)

The mean number of nesting females observed inside the nesting units after the application was calculated from the 3 highest numbers recorded during the assessments after application. The 3 highest abundances were taken for the calculations to avoid an underestimation of nesting females, because the number of females spending the night inside the cavities naturally decreases during the course of a study (when the cavities are filled up with cells, meaning there is no more space for females to sit inside the cavities).

#### Flight activity

For comparisons of flight activity, the first assessment after the application day was compared, and data were normalized to a time window of 3 min.

#### Cell production/reproductive performance

The total number of cells or cocoons/nesting unit was divided by the mean number of nesting females after application (see explanation of in the previous section, *Nest occupation (nesting activity*).

### Calculations and statistics

For comparability of the data between laboratories, only parameters that were assessed in all studies and with the same method are presented (see the *Data preparation* section; some laboratories recorded additional data, which are not shown in the present study).

Mean values and standard deviations of the respective parameters were calculated for each treatment group and assessment day/assessment period.

Statistical analysis was conducted on data from studies meeting the quality criteria. The statistical software program SAS® Ver 9.3 (2002–2010) was used for the statistical analysis. To analyze the potential impact of exposure to the test item, the data (nest occupation, flight activity, cell and cocoon production, and offspring production [emergence success, sex ratio, and bee weight]) were analyzed using pairwise tests. Statistical pretests to assess the normality and homoscedasticity of the data were performed prior to the actual statistical tests: Normality of the data was tested using the Shapiro–Wilk test (*p* ≤ 0.05), and homoscedasticity of data was tested using the *F* test (*p* ≤ 0.05). For data that were normally distributed and showed homogeneity of variance, a pooled *t* test (*p* ≤ 0.05) was conducted. If the data were normally distributed but not homoscedastic, a Satterthwaite *t* test was performed (*p* ≤ 0.05). Data that were not normally distributed were tested using a Mann–Whitney exact test (*p* ≤ 0.05). One‐sided tests were conducted (left‐sided [lower] for nest occupation, cell and cocoon production, emergence success, and weight data; right‐sided [upper] for immature mortality). To compare the establishment at the nesting units, a 2‐sided test was conducted. For flight activity and sex ratio data, 2‐sided tests were also performed.

The relationship between minimum temperatures and the percentage of released females establishing successfully at the nesting units before the application was examined using Spearman rank correlation.

The minimum detectable difference (MDD) defines the difference between the means of a treatment and the control that must exist to detect a statistically significant effect (Environment Canada 2005). The MDD can be calculated a posteriori for the statistical method used (e.g., *t* test), considering the actual test design (replication, selected type‐I error level alpha) and the sample variation. The absolute MDDs $\left({\text{MDD}}_{\text{abs}}\right)$ and the MDD relative to control means $\left({\text{MDD}}_{\%}\right)$ were calculated following Brock et al. (2015):

$${\mathrm{MDD}}_{\text{abs}}=t_{\left((,\alpha ,N-k,k,)\right)}\sqrt{\frac{{2s}^{2}}{n}}$$

where *s* represents standard error, square root of within‐group‐mean squares (variance), calculated from the pooled variance of all treatment groups; and $t_{\left(\alpha ,N-k,k\right)}$ is the tabulated critical value for the *t* test, considering the type‐I error α, the number of treatment groups *k* (including the control), and the *df* is the number of overall observations, with *N* − number of groups *k*.

$${\mathrm{MDD}}_{\%}=\frac{{\mathrm{MDD}}_{\text{abs}}}{{\overset{®}{x}}_{\text{control}}}\times 100$$

where ${\overset{®}{x}}_{\text{control}}$ is the control mean.

## RESULTS

With *O. bicornis*, a total of 15 studies (8 in 2016 and 7 in 2017) were performed using dimethoate as a test item, 4 with diflubenzuron (2 in 2016 and 2 in 2017), and 2 with fenoxycarb (in 2016). With *O. cornuta*, 2 studies were performed with dimethoate and 2 studies with diflubenzuron (2 in 2016 and 2 in 2017). An overview of all studies is given in Table 1.

### Studies that used dimethoate as a test item

**Figure 2:**
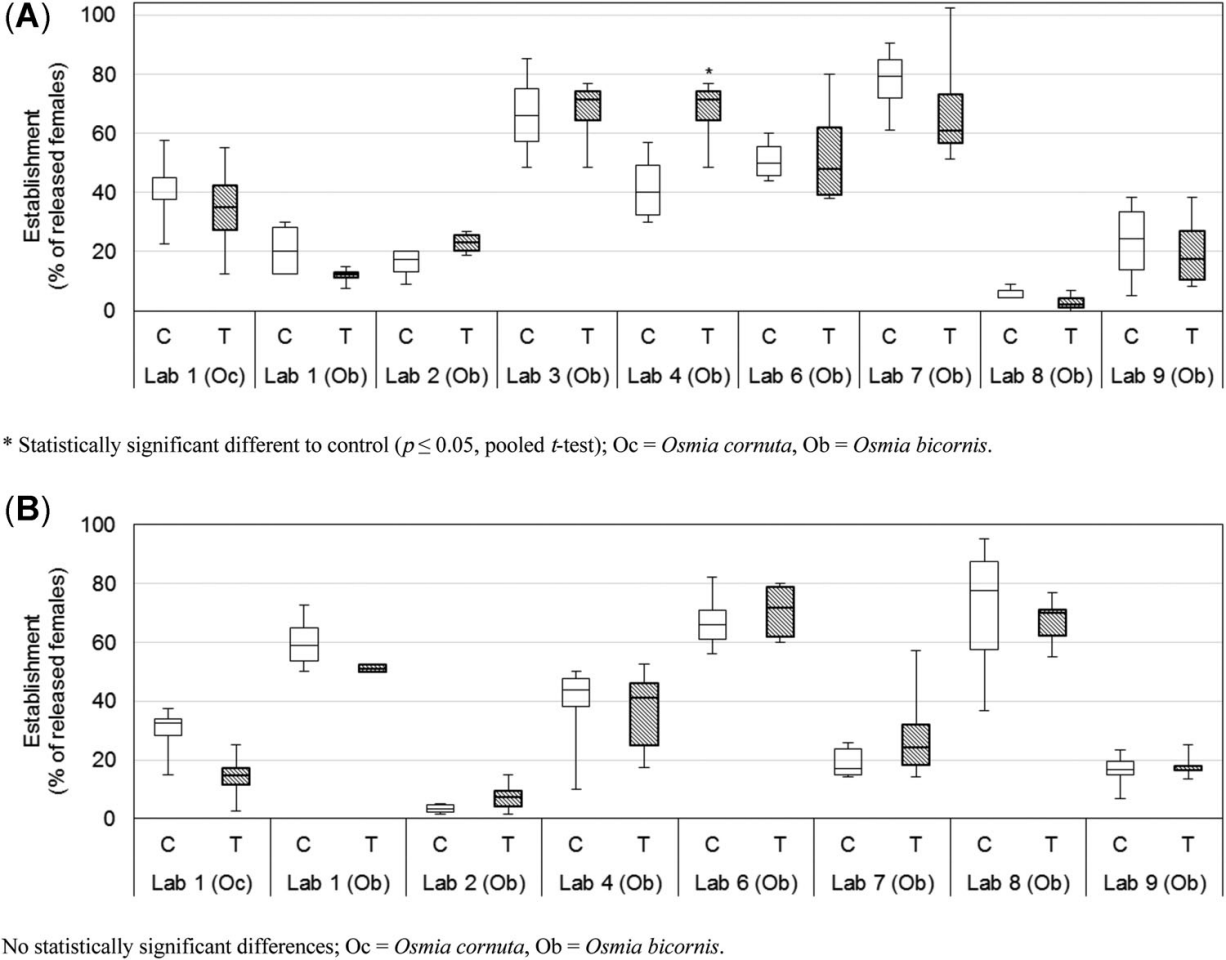
Boxplot of the percentage of released female bees nesting at the time of application in the ring test with the test items dimethoate and the bee species *Osmia bicornis* and *Osmia cornuta*. The median and 1st and 3rd quartile are presented; whiskers represent minimum and maximum values. For quality criteria, it was defined that a study was regarded valid if ≥30% of released females were found to occupy the nesting units in the night/morning before the application. (**A**) Data from 2016: 4 replicates/treatment group C and T (except for Lab 8: 3 replicates only). Lab 3, Lab 6, and Lab 7 are valid. (**B**) Data for 2017: 6 replicates/treatment group C and T (except for Lab 1: 4 replicates only). Lab 1, Lab 4, Lab 6, and Lab 8 are valid.

*Quality criteria*. In 2016, 4 of the 9 studies performed with dimethoate met the quality criteria (establishment), whereas the remaining 5 encountered problems (Table 1). These problems were either 1) a low or dissimilar establishment of female bees (nest occupation; Figure 2) between treatments before the application, or 2) the application was conducted too early with no flight activity present. In addition, the material used for the nesting units was found to be a factor influencing establishment, with a statistically significant lower establishment rate of females observed in plastic units compared with wooden units (*p* ≤ 0.05, pooled *t* test). Accordingly, the protocol was adjusted, and recommendations were made to use only medium‐density fiberboard trays in the ring test in 2017.

In 2017, 4 of the 8 studies performed with dimethoate met the quality criteria, and 4 studies did not (Table 1 and Figure 2). Due to low minimum temperatures in spring, the establishment of females before application was low in these trials and was strongly correlated to the minimum temperatures encountered at the time of these studies, with better establishment at higher temperatures (Spearman's correlation coefficient *r *= 0.83, *n *= 6, *p* < 0.05). However, some of these studies also used plastic trays, in which establishment rates were significantly lower compared with medium‐density fiberboard trays (data of 2016 and 2017 combined: *p* ≤ 0.05, pooled *t* test). In addition, one laboratory that performed 2 studies that met the quality criteria in 2016 could not meet these criteria in 2017. As an overall result, only 8 of the dimethoate studies (4 of 2016 and 4 of 2017) were analyed further. Of these 8 studies, 7 studies were conducted with *O. bicornis* and 1 study was conducted with *O. cornuta*. The results for both species are discussed together, because there is only one *O. cornuta* study, which, even though it used a different species, was always within the range of the performed *O. bicornis* studies.

#### Nest occupation

**Figure 3:**
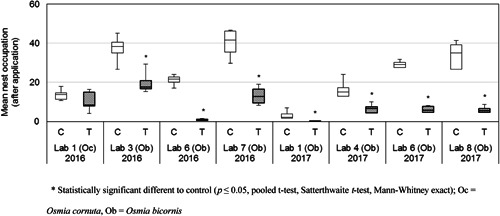
Boxplot of the mean number of nesting female bees (mean of 3 highest numbers) after the application of 75 g dimethoate/ha in all valid studies in the ring test with the bee species *Osmia bicornis* and *Osmia cornuta*. The bees were counted in the nesting units at night or early in the morning. In 2016, 4 replicates and in 2017, 6 replicates (except for Lab 1: 4 replicates only) were used per treatment group C and T.

In 7 of the 8 valid studies mean nest occupation was significantly lower after the application of dimethoate in the treated tunnels compared with control tunnels (*p* ≤ 0.05, pooled *t* test, Mann–Whitney exact, Satterthwaite *t* test; Table 2 and Figure 3). The significant reductions in mean nest occupation in the dimethoate‐treated tunnels ranged from 46.1 to 97.4% compared with the control.

**Table 2:** Data overview of the ring‐test results with the test item dimethoate and the bee species *Osmia cornuta* and *Osmia bicornis* (mean ± standard deviation)

		Laboratory							
		1		3	4	6		7	8
Parameter	Control/treatment	2016^a^	2017^b^	2016^b^	2017^b^	2016^b^	2017^b^	2016^b^	2017^b^
Nest occupation (nesting activity)	C	13.5 ± 3.1	3.2 ± 2.6	37.1 ± 7.6	14.6 ± 6.9	21.1 ± 3.0	28.5 ± 2.8	39.9 ± 8.0	32.1 ± 9.6
	T	10.3 ± 5.2	0.1^d^, * ± 0.2	20.0^c^, * ± 6.3	6.1^c^, * ± 3.0	0.8^e^, * ± 0.4	5.9^c^, * ± 1.9	13.3^c^, * ± 5.0	5.6^e^, * ± 2.1
Flight activity (/3 min)	C	n.a.	15.8 ± 3.8	10.8 ± 5.2	5.0 ± 2.5	1.0 ± 1.4	17.7 ± 2.9	7.9 ± 3.3	13.3 ± 2.3
	T	n.a.	0.5^e^, * ± 0.6	0.7^d^, * ± 0.5	2.1^c^, * ± 1.4	0.0 ± 0.0	6.2^c^, * ± 4.3	0.4^e^, * ± 0.8	2.2^c^, * ± 1.7
Cell production (/female)	C	10.7 ± 2.5	14.1 ± 7.9	12.8 ± 2.1	14.1 ± 2.0	11.1 ± 2.5	10.2 ± 1.6	11.0 ± 3.8	23.8 ± 3.3
	T	7.3 ± 4.0	0.8^e^, * ± 1.5	6.9^c^, * ± 1.6	5.2^c^, * ± 1.1	8.3 ± 4.7	3.9^c^, * ± 1.2	3.7^e^, * ± 0.9	t24.6 ± 8.7
Cell production (/nesting unit)	C	149.0 ± 60.5	39.8 ± 27.4	483.5 ± 151.9	214.3 ± 110.7	230.3 ± 36.6	288.5 ± 33.4	289.0 ± 25.4	739.5 ± 143.8
	T	65.2^c^, * ± 20.5	1.0^e^, * ± 0.8	138.5^c^, * ± 56.3	33.0^e^, * ± 18.5	5.5^e^, * ± 3.1	23.2^e^, * ± 10.7	31.5^c^, * ± 24.0	125.7^d^, * ± 30.4
Cocoon production (/female)	C	10.0 ± 2.7	13.2 ± 8.3	12.8 ± 2.1	13.0 ± 1.8	6.9 ± 2.0	9.1 ± 1.5	n.a.	20.6 ± 2.4
	T	6.6 ± 3.8	3.0 ± n.a.	6.9^c^, * ± 1.6	4.3^c^, * ± 1.1	5.1 ± 3.2	3.1^c^, * ± 1.2	n.a.	13.4^c^, * ± 4.6
Cocoon production (/nesting unit)	C	139.0 ± 59.0	36.0 ± 23.1	456.5 ± 146.2	197.7 ± 101.5	141.0 ± 24.9	256.8 ± 28.3	n.a.	656.2 ± 123.0
	T	58.8^c^, * ± 27.1	1.0^e^, * ± 0.8	130.0^c^, * ± 49.9	26.2^e^, * ± 13.9	3.3^d^, * ± 1.0	18.5^e^, * ± 9.3	n.a.	71.3^d^, * ± 28.2
Emergence success (%)	C	66.6 ± 4.1	91.6 ± 4.3	82.5 ± 4.4	84.4 ± 11.4	97.5 ± 0.8	73.2 ± 5.2	93.5 ± 9.1	94.5 ± 3.4
	T	77.5 ± 7.2	100.0 ± n.a	85.3 ± 4.6	89.9 ± 9.8	93.8 ± 12.5	83.1 ± 13.6	89.2 ± 12.8	92.3 ± 5.9
Sex ratio (male:female)	C	1.8 ± 0.6	1.3 ± 0.9	3.0 ± 0.5	2.6 ± 1.1	3.8 ± 1.9	9.5 ± 4.1	5.3 ± 2.0	2.3 ± 0.4
	T	2.0 ± 1.0	0.3 ± 0.6	12.8^d^, * ± 10.3	3.9 ± 2.2	0.8^e^, * ± 0.3	4.4^d^, * ± 0.7	3.7 ± 3.1	9.4^d^, * ± 11.2
Female offspring weight (mg)	C	146.5 ± 6.1	113.5 ± 11.9	84.7 ± 6.6	99.3 ± 7.4	86.0 ± 11.2	88.4 ± 6.6	96.7 ± 0.8	95.0 ± 4.7
	T	138.7 ± 10.1	107.9 ± 12.4	89.4 ± 16.7	92.0 ± 12.5	76.8 ± 11.8	83.4 ± 24.5	100.6 ± 4.1	79.4^c^, * ± 6.2
Male offspring weight (mg)	C	84.9 ± 2.8	56.8 ± 6.2	55.1 ± 1.4	57.0 ± 2.4	59.7 ± 3.5	58.2 ± 2.2	55.6 ± 2.0	57.8 ± 3.0
	T	82.2 ± 4.1	73.7 ± n.a.	48.8^c^, * ± 2.4	56.5 ± 2.7	64.7 ± 18.4	54.0^c^, * ± 3.9	41.1 ± 19.7	51.2^c^, * ± 3.1

^a^ Study with *Osmia cornuta*.

^b^ Study with *Osmia bicornis*.

^c^ Pooled *t*‐test.

^d^ Mann–Whitney exact.

^e^ Satterthwaite *t*‐test.

* *p* ≤ 0.05.

C = control; T = treatment; n.a. = not assessed; nest occupation = mean of 3 highest no. after application; flight activity = first assessment after application; cell production/female = no. of cells/mean of 3 highest no. of nesting females; cell production/nesting unit = no. of all cells/nesting unit; cocoon production/female = no. of cocoons/mean of 3 highest no. of nesting females; cocoon production/nesting unit = no. of all cocoons/nesting unit; emergence success = % of bees successfully emerging from cocoons after overwintering; sex ratio = sex ratio of bees emerging from cocoons after overwintering; female and male offspring weight = mean weight of female and male bees emerging from cocoons after overwintering.

#### Flight activity

**Figure 4:**
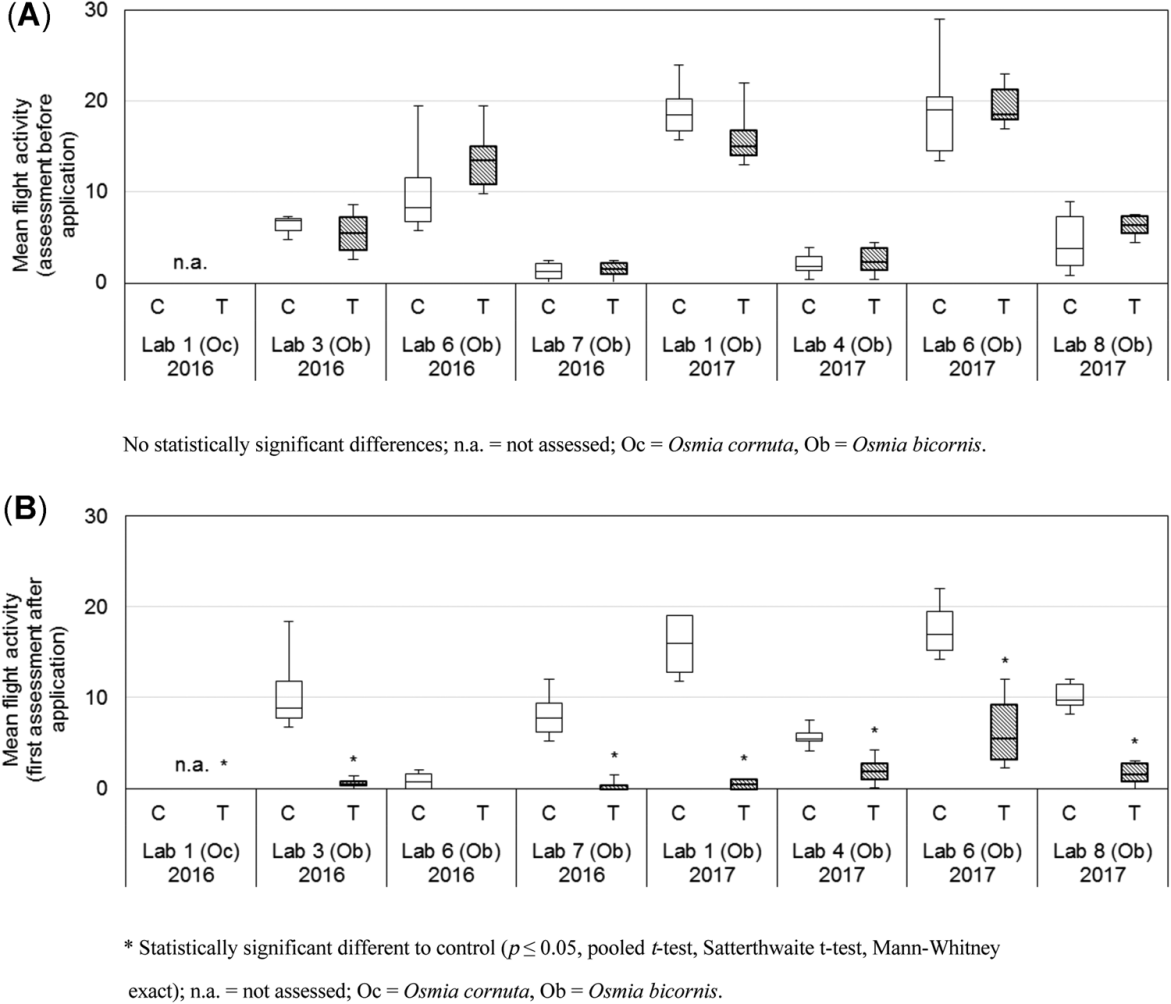
Boxplot of the mean flight activity of female bees (entering the nesting unit in 3 min) at the first assessment after the application of 75 g dimethoate/ha in all valid studies in the ring test with *Osmia bicornis* and *Osmia cornuta*. In 2016, 4 replicates and in 2017, 6 replicates (except for Lab 1: 4 replicates only) were used per treatment group C and T.

Flight activity at the nesting units was significantly reduced in 6 of 7 studies after the application (no data for laboratory 1 (2016) available; *p* ≤ 0.05, pooled *t* test, Mann–Whitney exact, Satterthwaite *t* test; Figure 4). In that study, in which no significant difference was found, the flight activity was low on the assessment day with 1.0 (±1.4 standard deviation [SD]) in the control group compared with 0.0 (±0.0 SD) in the treated group. The observed significant reductions in flight activity ranged from 54.1 to 96.8%.

#### Reproduction

**Figure 5:**
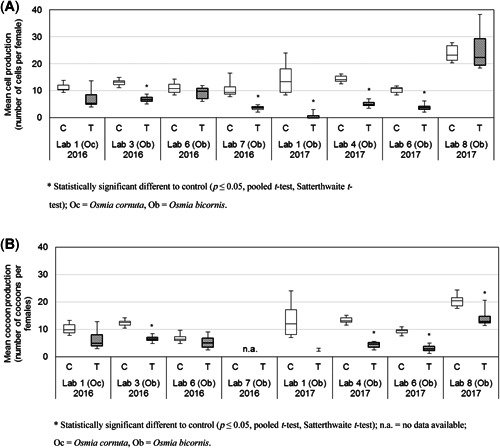
Boxplot of the mean number of cells (**A**) and cocoons (**B**) produced/female bee (calculated based on the mean of the 3 highest numbers counted during nest occupation assessments) after the application of 75 g dimethoate/ha in all valid studies in the ring test with *Osmia bicornis* and *Osmia cornuta*. In 2016, 4 replicates and in 2017, 6 replicates (except for Lab 1: 4 replicates only) were used per treatment group C and T.

The results indicated a reproductive performance under semifield conditions of approximately 0.4 to 1.5 cells/nesting female/d in the control, if weather conditions were suitable for foraging (no rain or strong wind and temperatures >10 °C). The total cell and cocoon production/nesting unit were both significantly lower in the dimethoate treatment in all studies (*p* ≤ 0.05, pooled *t* test, Mann–Whitney exact, Satterthwaite *t* test). The total number of cells and cocoons was reduced by 56.5 to 97.6% and 57.7 to 96.5%, respectively. The number of cells produced/nesting female was significantly lower in the dimethoate‐treated tunnels compared with the control in 5 of 8 studies (*p* ≤ 0.05, pooled *t* test, Satterthwaite *t* test; Figure 5). The significant reductions ranged from 46.2 to 94.7%. Regarding cocoon production, the number of cocoons/female was significantly reduced in 4 of 7 studies (no data for laboratory 7 (2016) available; *p* ≤ 0.05, pooled *t* test, Satterthwaite *t* test; Figure 5). The reductions ranged from 34.8 to 94.3% in the toxic reference compared with the control.

#### Offspring production

For emergence success no statistically significant differences between the treatment and the control were observed in any of the studies. Concerning the mean female and male weights as well as the sex ratio, the results were mixed. In 1 of the 8 studies females were significantly lighter in the dimethoate treatment compared with the control (*p* ≤ 0.05, pooled *t*‐test), whereas males were found to be lighter in 3 of the 8 studies (*p* ≤ 0.05, pooled *t* test). The sex ratio was significantly different in the treatment compared with the control in half of the studies, with a male‐biased ratio in 2 of the 8 studies and a female‐biased ratio in 2 of the 8 studies (*p* ≤ 0.05, Mann–Whitney exact, Satterthwaite *t* test).

#### MDDs

The MDD values for the different parameters provide an indication of the variability of the respective parameters and the statistical significance of effects, which can be determined (Table 3). In 2017 the number of replicates/treatment was increased from 4 to 6, to decrease variation and to decrease the associated MDD values. Increasing the number of replicates from 4 to 6 did improve MDD values for flight activity and mean male offspring weight, but had no further impact on the MDD values for other parameters. Combining the results of both years, the MDDs were highest for the parameter flight activity, with a mean MDD of 59.1, which means that a difference of 59.1% from controls can be detected statistically. The parameters mean female offspring weight and mean male offspring weight had the lowest mean MDDs, at 13.2 and 14.8, respectively. Based on the MDD classes proposed in the EFSA Aquatic Guidance Document (European Food Safety Authority Panel on Plant Protection Products and Their Residues 2013; Supplemental Data, Table SI‐2), small effects could be detected in all studied parameters with MDDs below 50%, with the exception of sex ratio and flight activity, for which only medium effects could be detected (MDDs of 50–70%). Regarding the robustness of the parameters over multiple studies, indicated by a small range in MDD values, the most reliable parameters were mean female offspring weight followed by nest occupation, mean male offspring weight, and total cocoon production.

**Table 3:** Minimum detectable differences (MDDs) for different parameters assessed in the ring test with the test item dimethoate and the bee species *Osmia cornuta* and *Osmia bicornis*

Parameter	Mean			Range
Year	2016	2017	2016/2017	2016/2017
No. of studies	4	4	8	8
Nest occupation	33.2	40.3	36.7	35.4
Flight activity (/3 min)	70.1	50.9	59.1	64.5
Cell production (/female)	35.8	33.0	34.4	53.7
Total cell production	41.4	43.6	42.5	45.5
Cocoon production (/female)^a^	35.8	34.8	35.2	52.7
Total cocoon production^a^	33.7	46.7	41.1	38.9
Emergence success (%)	13.1	24.9	19.0	70.4
Sex ratio (male:female)	50.5	56.4	53.4	51.5
Female offspring weight (mg)	11.8	14.6	13.2	16.2
Male offspring weight (mg)	19.4	10.2	14.8	37.5

^a^ For 2016, only data from 2 laboratories are available.

For definitions of the parameters, see Table 2.

### Studies that used diflubenzuron as a test item

Of the total 6 studies conducted with diflubenzuron 3 were considered valid (Table 1). In 2 of the valid studies total immature mortality was significantly higher in the diflubenzuron treatment compared with the control (*p* ≤ 0.05, pooled *t* test; Table 4). In the third valid study the immature mortality was not significantly higher in the diflubenzuron treatment when the whole exposure period was examined. However, a significantly higher mortality of eggs and larvae was found for cells built between 0 and 3 d after the application and between 3 and 6 d after the application (*p* ≤ 0.05, Mann–Whitney exact, pooled *t* test).

**Table 4:** Data overview of the ring‐test results with the test item diflubenzuron and the bee species *Osmia cornuta* and *Osmia bicornis* (mean ± standard deviation)

		Laboratory		
		1		4
Parameter	Treatment/control	2016^a^	2017^b^	2017^b^
Nest occupation (nesting activity)	C	13.7 ± 2.9	3.2 ± 2.6	14.6 ± 6.9
	T	15.4 ± 4.0	2.0 ± 1.0	15.8 ± 6.8
Flight activity/3 min)	C	n.a.	15.8 ± 3.8	5.0 ± 2.5
	T	n.a.	12.3 ± 2.2	3.3 ± 1.7
Cell production (/female)	C	10.7 ± 2.5	14.1 ± 7.9	14.1 ± 2.0
	T	7.3 ± 4.0	16.4 ± 5.9	15.0 ± 0.6
Cell production (/nesting unit)	C	149.0 ± 56.5	39.8 ± 27.4	214.3 ± 110.7
	T	133.4 ± 53.6	34.8 ± 23.7	235.0 ± 93.3
Cocoon production (/female)	C	10.0 ± 2.7	13.2 ± 8.3	13.0 ± 1.8
	T	7.0 ± 3.0	8.6 ± 2.9	11.8 ± 1.1
Cocoon production (/nesting unit)	C	139.0 ± 59.0	36.0 ± 23.1	197.7 ± 101.5
	T	102.8 ± 43.6	17.8 ± 10.9	180.5 ± 60.1
Immature mortality (%)/nesting unit)	C	5.9 ± 4.4	8.2 ± 6.3	7.5 ± 1.0
	T	61.2* ^,c^ ± 4.5	46.0* ^,c^ ± 5.8	22.1 ± 5.4^d^
Emergence success (%)	C	66.6 ± 4.1	91.6 ± 4.3	84.4 ± 11.4
	T	71.0 ± 15.4	96.6 ± 3.6	78.4 ± 8.0
Sex ratio (male:female)	C	1.8 ± 0.6	1.3 ± 0.9	2.6 ± 1.1
	T	2.2 ± 0.7	1.8 ± 1.3	2.6 ± 0.5
Female offspring weight (mg)	C	146.5 ± 6.1	113.5 ± 11.9	99.3 ± 7.4
	T	153.0 ± 13.3	99.6 ± 14.5	96.6 ± 4.8
Male offspring weight (mg)	C	84.9 ± 2.8	56.8 ± 6.2	57.0 ± 2.4
	T	86.1 ± 5.4	61.6 ± 21.2	58.1 ± 0.9

^a^ Study with *Osmia cornuta*.

^b^ Study with *Osmia bicornis*.

^c^ Pooled *t* test.

^d^ Although no effect was seen on immature mortality for the whole exposure period, a significant increase in mortality was observed for the period 0 to 3 d after application (mean C: 11.1%, T: 84.3%) and 3 to 6 d after application (mean C: 1.8%, T: 17.4%).

* *p* ≤ 0.05.

For definitions of the parameters, see Table 2; immature mortality = difference of cell and cocoon production.

### Studies that used fenoxycarb as a test item

Both studies with fenoxycarb were valid (Table 1). No statistically significant differences in the fenoxycarb treatment compared with the control were found for any of the studied parameters (nest occupation, flight activity, cell and cocoon production, and immature mortality). One of the laboratories found a significantly increased immature mortality for cells produced between the day before the application and 2 d after application, but not in the subsequent intervals (see also Lückmann et al. 2018).

## DISCUSSION

The results of the ring test summarized in the present study were collected using *O. bicornis* and *O. cornuta* as test organism. The 2 crops used in the studies, winter oilseed rape (*B. napus*) and purple tansy (*P. tanacetifolia*), were both highly attractive to mason bees and provided enough nectar and pollen for the production of offspring.

The assessed parameters were chosen to be able to detect both sublethal (e.g., a reduced flight activity) and lethal effects on adult bees and their offspring and to fit the specific life‐history traits of solitary bees. According to the calculated MDDs small effects (defined as MDD values < 50%) could be determined statistically for all studied parameters, with the exception of sex ratio and flight activity (medium effects only). It should be noted that these effect classes are based on those published in the EFSA Aquatic Guidance Document (European Food Safety Authority Panel on Plant Protection Products and Their Residues 2013) and have not yet been validated for pollinator studies. Concerning the number of replicates, 4 replicates are sufficient because the MDD values of most endpoints did not improve when the number of replicates was increased to 6. However, the endpoints flight activity and mean male offspring weight were exceptions and did improve.

The results of the ring test show that once a study was valid (i.e., >30% of nesting females present at the time of application), it could detect small effects (<50% from controls) on the endpoints offspring (emergence rate and offspring weight) and the reproducing units female bees (nest occupation and cell/cocoon production/female) compared with controls. The results from the ring test also show that despite the natural variation present when working with *Osmia*, the observed effect sizes can sometimes be small, because the lowest MDD values observed were between 8 and 14%. This is even better than for honeybee endpoints, for which MDD values of 10 to 15% were observed (Candolfi et al. 2018). However, for most endpoints, higher MDDs were found than those found for honeybees (Candolfi et al. 2018).

It has to be taken into consideration that some of the participating laboratories did not have any hands‐on experience with this test organism prior to the ring test. The principle of the first year of ring testing was to follow a general protocol but allow participating laboratories to experiment and find the best practice to conduct the studies. In the second year the protocol was more detailed and prescriptive to standardize the test. Because semifield studies are complex and also depend on external factors, such as weather conditions, it is not easy to establish a new test system. Each laboratory first needs to gain some experience with the handling of the test organism before they are able to conduct valid studies. This was also seen in the ring test. In the first year approximately 50% of laboratories conducted at least one valid study, which increased to approximately 60% in the second year. The biggest issue was a sufficient establishment of females in the nesting units at test start (quality criterion for a valid study). The use of less attractive nest material and cold spring temperatures were identified as severe challenges. The medium‐density fiberboard trays were found to be more suitable than other types of nesting material for tests with *O. bicornis*. Concerning the problem with cold temperatures in spring, one solution could be to offer an insulated retreat for the bees during cold nights, for example, by insulating the nesting units. A second option could be to conduct studies using early sown *Phacelia*. The experience from some laboratories is that studies using *Phacelia* conducted later than June are not recommendable because immature mortality can be quite high, presumably due to the senescence of females and potentially high temperatures in midsummer. This also correlates with the natural occurrence of *O. bicornis* from March to June.

Regarding the assessed parameters the following points have to be considered, when conducting a semifield study with *O. bicornis* or *O. cornuta*. The right timing of the application is important, because it influences the parameter sex ratio. Female brood is produced first followed by male brood, meaning that the number of female brood produced after application is low when the application is conducted too late and female bees have already switched to laying mainly unfertilized, male eggs (because only exposed eggs, which were laid after the application, are assessed). It is also known that the sex ratio is influenced by the availability of food resources and the season in, for instance, *O. lignaria* (Torchio and Tepedino 1980). More males are produced when resources are scarce or late in the season, when parental fitness is lower due to senescence of the parental females, because male offspring cost less resources to produce than female offspring. This emphasizes the importance of providing enough flower resources in a semifield tunnel and conducting studies within the natural season of the test species. For instance, a high density of parental females of 2.2/m² resulted in a strongly male‐biased sex ratio in a semifield study conducted with *O. cornuta* (Strobl et al. 2019).

Concerning flight activity, the observation time should ideally be longer than the 3 min specified in the protocol, because the mean duration of one foraging trip of *O. bicornis* is approximately 12 min (Gathmann and Tscharntke 2002). However, no data for the foraging trip duration exist in semifield tunnels. Nonetheless, longer observation times would surely decrease the variability between replicates because flight activity strongly depends on the current weather conditions and thus on the timing of the assessments. However, because currently no electronic data collection (such as video recording with count software) is available, it would be difficult to increase the observation time due to the time and people required to conduct manual counts. Moreover, increasing the observation time increases the probability of multiple counts of the same females.

Another point of discussion within the ring‐test group was the normalization of reproduction data. It was decided that it is necessary to normalize values for comparisons between treatment groups or studies and to cancel out variation between replicates caused by differences in the number of nesting females. Two different approaches were proposed: to use the maximum number or the mean number of nesting females to calculate the number of cells or cocoons produced by a female during the study. Both are possible, but when using a mean value, nest occupation data from the beginning and end of the study have to be excluded. Naturally, these values are low at the beginning of the establishment period and at the end of the study, when females die at the end of their life span (~4 wk). Thus, including these values in the calculation of the mean value would lead to an underestimation of active females during the study and an overestimation of reproductive performance. Our proposal is to use the 3 highest values recorded during nest occupation assessments for the calculation of a mean number. This would cover the peak in nesting activity and be more robust than using a single value.

Regarding the tested reference item dimethoate, all valid studies with *O. bicornis* found statistically significant reductions in nesting activity and cell or cocoon production/nesting unit due to adult mortality following its application at a rate of 75 g a.i./ha. In the valid study with *O. cornuta*, however, no significant reduction in nesting activity was observed. A possible explanation is that *O. cornuta* is slightly bigger than *O. bicornis* and therefore might be less susceptible to dimethoate The application rate of dimethoate has to be increased in studies with *O.cornuta*, to cause significant effects on nesting activity. Because currently only one valid study was available for *O. cornuta*, further research is needed to give a clear recommendation on the application rate of dimethoate in such studies. The results for total cell and cocoon production were more consistent, showing significant reductions in all studies This is in contrast to cell and cocoon production/nesting female, which was not significantly reduced in all studies because the surviving females still produced a considerable number of eggs after the application in some studies. Statistically significant effects of dimethoate on flight activity were also shown in all studies, except for one study in which flight activity was too low in the control to compare the data with the treatment due to bad weather conditions. The analysis of offspring production delivered mixed results. Dimethoate did not have an effect on emergence success in any of the studies, but in some studies a statistically significant reduction in male bee weights was found. The sex ratios were generally very variable between studies, ranging from 1.3 to 9.5 (male:female) in the controls.

The second tested toxic reference item, diflubenzuron, had statistically significant effects on brood development, causing mortality of eggs and larvae at a rate of 200 and 216 g a.i./ha, respectively. Because these findings are limited to the results of 2 small additional studies, effects of this potential reference item should be tested further. Regarding the second insect growth regulator tested, fenoxycarb, no significant effects were observed at rates of 300 and 600 g a.i./ha in the studied parameters. In a study (not part of the ring test, but following the ring‐test protocol) conducted in 2015 (with 150 and 350 g a.i./ha), a small but statistically significant increase in brood mortality was observed at both rates (Knäbe et al. 2016). This may indicate that fenoxycarb does cause some brood mortality, but only directly after the application. Because each cell is provisioned with nectar and pollen within 1 or 2 d and then closed, the concentration of residues varies from cell to cell depending on the date of provisioning in relation to the application. This assumption is supported by the results of Lückmann et al. (2018), who observed an increased brood mortality within the first 2 d after application but not later. Therefore it is recommended to evaluate nest occupation, cell production, and the development of the brood in 3‐d intervals to allow the analysis of time‐dependent effects due to decreasing exposure.

In summary, once >30% females have established, dimethoate is a reliable positive control at the tested rate of 75 g a.i./ha, displaying acute effects such as reduction of flight activity, increase in adult mortality (shown by nest occupation), and reproduction ability of the females (total cell and cocoon production). On the other hand, no effects on larval and pupal development were observed. The application rate of 75 g a.i./ha is lower than the rate used in tunnel studies with *A. mellifera*, in which 400 g a.i./ha dimethoate is applied as the toxic reference (Commission des Essais Biologiques 2011). The effects seen in *Osmia* sp. at lower application rates may be linked to differences in life‐history traits. As each *Osmia* female is reproducing, the death of an individual female is directly affecting the reproductive performance of the population. In contrast, the reproductive output of a honey bee colony will not be reduced by the death of a single worker bee. Social bee species possess a certain buffer capacity, which solitary species lack (Sgolastra et al. 2018). This underlines the necessity to account for differences in life‐history traits between solitary and social bee species in the risk assessment of plant protection products that pose a risk to bees (i.e., insecticides).

In contrast to dimethoate, effects on the brood (i.e., immature mortality) were found using the insect growth regulator diflubenzuron as the test item. More studies should be conducted to confirm these results and the application rate of diflubenzuron, because the data set for diflubenzuron is limited. Also, with regard to the uncertain availability of well‐established toxic reference items such as dimethoate in Europe and globally, other active substances may be considered. However, they should be tested beforehand to establish the application rates and type and magnitude of effects that can be expected.

## CONCLUSIONS

In conclusion, the ring‐test protocol proved to be adequate once the study comprised a well‐established population of female *Osmia* bees, and the results improved in the second year as the laboratories increased their experience with the test organism. It is noted that the success of a study strongly depends on the experience of the experimenter, on the crop quality, the quality of the cocoons, and the weather conditions.

It was shown that studies with *O. bicornis* and *O. cornuta* in winter oil seed rape (*B. napus)* or purple tansy (*P. tanacetifolia*) are feasible. Of the assessed parameters, nesting activity, flight activity, and total cell and total cocoon production were the most robust. Dimethoate can be used as a toxic reference item at a rate of 75 g a.i./ha in studies with *O. bicornis*. However, a higher rate might be needed in studies with *O. cornuta*. Further research is also needed to identify reliable insect growth regulator reference items and respective application rates, because the data set on diflubenzuron was very limited. Based on the experiences in the ring test, recommendations for a semifield study design with *O. bicornis* and *O. cornuta* were summarized and are provided in the Supplemental Data.

## Supplemental Data

The Supplemental Data are available on the Wiley Online Library at https://doi.org/10.1002/etc.4874.

## Supporting information

This article includes online‐only Supplemental Data.

Supporting information.

## Data Availability

Data, associated metadata, and calculation tools are available from the corresponding author (leafranke@eurofins.com).
