# Supplementary material for: Results of 2‐Year Ring Testing of a Semifield Study Design to Investigate Potential Impacts of Plant Protection Products on the Solitary Bees Osmia Bicornis and Osmia Cornuta and a Proposal for a Suitable Test Design
Source: Environ Toxicol Chem. 2020 Nov 16;40(1):236–50. doi: 10.1002/etc.4874 (PMC7839555; doi:10.1002/etc.4874)
Supplement: Supplementary file 1 — Supporting information. [file ETC-40-236-s001.docx]

Table SI-1. Recommendations for a semi-field study design for the assessment of plant protection product side effects on the solitary bee species *Osmia bicornis* and *Osmia cornuta*

| 1) PRINCIPLE DESIGN OF THE TEST METHOD | |
| --- | --- |
| The test consists of at least three treatment groups, a water treated or untreated control, one or more test item groups and the toxic reference item treatment group (applied with the active ingredient dimethoate or in case of insect growth regulators the active ingredient diflubenzuron). Mason bee cocoons or adults, e.g. *Osmia cornuta* or *Osmia bicornis*, have to be placed in the tunnel as soon as first flowers are open (approx. BBCH 59-60). Nesting units are placed in each tunnel where the bees will establish their brood nests. The adult bees and their larvae are exposed to the nectar and pollen of the crop throughout the flowering period. The potential effects on the reproduction ability and offspring production are the focal points of the test. After the end of the exposure period the development of the progenies is followed until the following spring, where the reproduction success is determined by the number of emerged individuals. | |
| 2) TEST METHOD  2.1) TEST SYSTEM / TEST ORGANISM | |
| Species/Variety | *Osmia bicornis* (Linnaeus, 1758)  *Osmia cornuta* (Latreille, 1805) |
| Source | Cocoons of the test species from a commercial supplier. |
| Storage and handling of test organism | - before incubation: storage of cocoons at approx. 1-4 °C - incubation (to synchronize emergence with flowering): at approx. 22 °C (± 2 °C) and a relative air humidity of approx. 60-80% - release in the field (start of study): cocoons or adult bees are placed in nesting units as soon as first flowers are open (approx. BBCH 59-60). - application: in case of a release as cocoons all remaining closed cocoons (from which no bees have emerged) have to be removed from the tunnels before the application. - end of flowering (BBCH 69, end of study): nesting units are covered with a fine gauze mesh (to stop nesting and to protect bee brood from parasites); the units are left in the field until a late stage of larval development is reached storage until harvest of cocoons: the trays can be carefully transported from the field site to a storage place (approx. 1-2 months after end of flowering); trays should be stored dry and at ambient temperatures - harvest of cocoons: maturation of bees in the cocoons has to be checked by opening several cocoons per treatment group (in autumn); cocoons have to be cleaned and dried before weighing (remove pollen and dust by rinsing with cold water (no detergent!)) - hibernation: cocoons are stored at approx. 1-4 °C and a relative air humidity of approx. 60-80% for approx. 4 months until incubation - incubation: subsamples of cocoons are incubated to assess emergence success (F1 generation); cocoons are incubated at approx. +22 C ± 2 °C and a relative air humidity of approx. 60-80%. |
| Description of nesting units/nesting material | The nesting units should consist of:   - outer chassis with rainproof roof - MDF (medium-density fiberboard) trays with a cavity diameter of 8 mm x 8 mm x 150 mm (*O. bicornis*) or 10 mm x 10 mm x 150 mm (*O. cornuta*) - trays covered with a transparent film to allow marking of cells - trays offering a provision of at least 2 cavities per female - placed above the ground (to avoid humid conditions) - oriented towards south-east (to enhance activity in the morning) - wet, loamy soil has to be provided by digging a hole and adding water or by providing a box etc. containing loamy soil (needed for construction of cell partitions and nest plugs) |
| 4) TEST DESIGN | |
| Test design | Semi-field study |
| Tunnel size | Min. 30 m², preferably 60 m² (max. 1.0 female per m²) |
| Duration of exposure phase | Approx. 3 to 6 weeks (until end of flowering), |
| Crop | Oil seed rape (*Brassica napus*), purple tansy (*Phacelia tanacetifolia*)  Note: the test should be performed as close as possible to the natural activity period of the test species (in spring or early summer) |
| Number of treatment groups | ≥ 1 test item treatment, 1 control (negative control), 1 toxic reference (positive control) |
| Replicates per treatment group | ≥ 4 |
| Toxic reference item | Dimethoate (at least 75 g a.i./ha); Diflubenzuron (at least 200 g a.i./ha) |
| Test organism per treatment group | One nesting unit per tunnel and release of ≥ 30 females per replicate and adapted males to a female:male sex ratio of 1:1.5 (♀ : ♂). |
| Application | - spray application (following Good Agricultural Practice); - the toxic reference should be applied during bee flight (as soon as at least 20 females are nesting per nesting unit and first cells are built) - all cells built before the application (all pollen/pollen mass in cavities, also without egg and/or mud wall) have to be marked on the transparent sheet |
| 5) QUALITY CRITERIA | |
| Parameters | At least a significant reduction in a parameter in the toxic reference compared to the control. |
| 6) ASSESSMENTS / PARAMETERS | |
| Emergence success (parental generation) | Counting the number of females and males emerged from the introduced cocoons (by counting empty cocoons) in 3- to 4-day-intervals. |
| Establishment/Nest occupation (nesting activity) | Counting the number of nesting females per nesting unit when there is no flight activity at night (after sun-set) or early in the morning (before sunrise) in 3- to 4-day-intervals and in parallel to the cell production assessments (see below). |
| Reproduction (cell and cocoon production) | Cell production:  Counting the number of cells (= pollen mass with egg and mud wall) produced after application in 3- to 4-day-intervals (=cohort). Either take pictures or mark produced cells with different coloured permanent markers on the transparent sheet.  Cocoon production:  Counting the number of developed cocoons in autumn before hibernation. If assessments on cell production were made in intervals, each cohort should be harvested individually (if the number of cocoons in a cohort is too low for statistical analysis cohorts can be pooled). |
| Flight activity | Counting the number of bees entering the nesting cavities at each nesting unit during a defined time interval (at least 5 minutes);  at least two assessments on the application day (one directly before and one shortly after the application) and one assessment on the first day after the application. Flight assessments should be conducted in parallel in the control and the test item treatment(s). |
| Parasitation | Check, count and remove parasites. |
| Emergence success (F1 generation) | Counting the number of females and males emerging from the subsamples of cocoons (only bees emerging from exposed cocoons (= produced after the application)); also differentiate between cohorts. Check for reason of remaining closed cocoons (not mature, parasitized, mature but dead).  OPTIONAL: Individual weighing of each emerged female and male. |


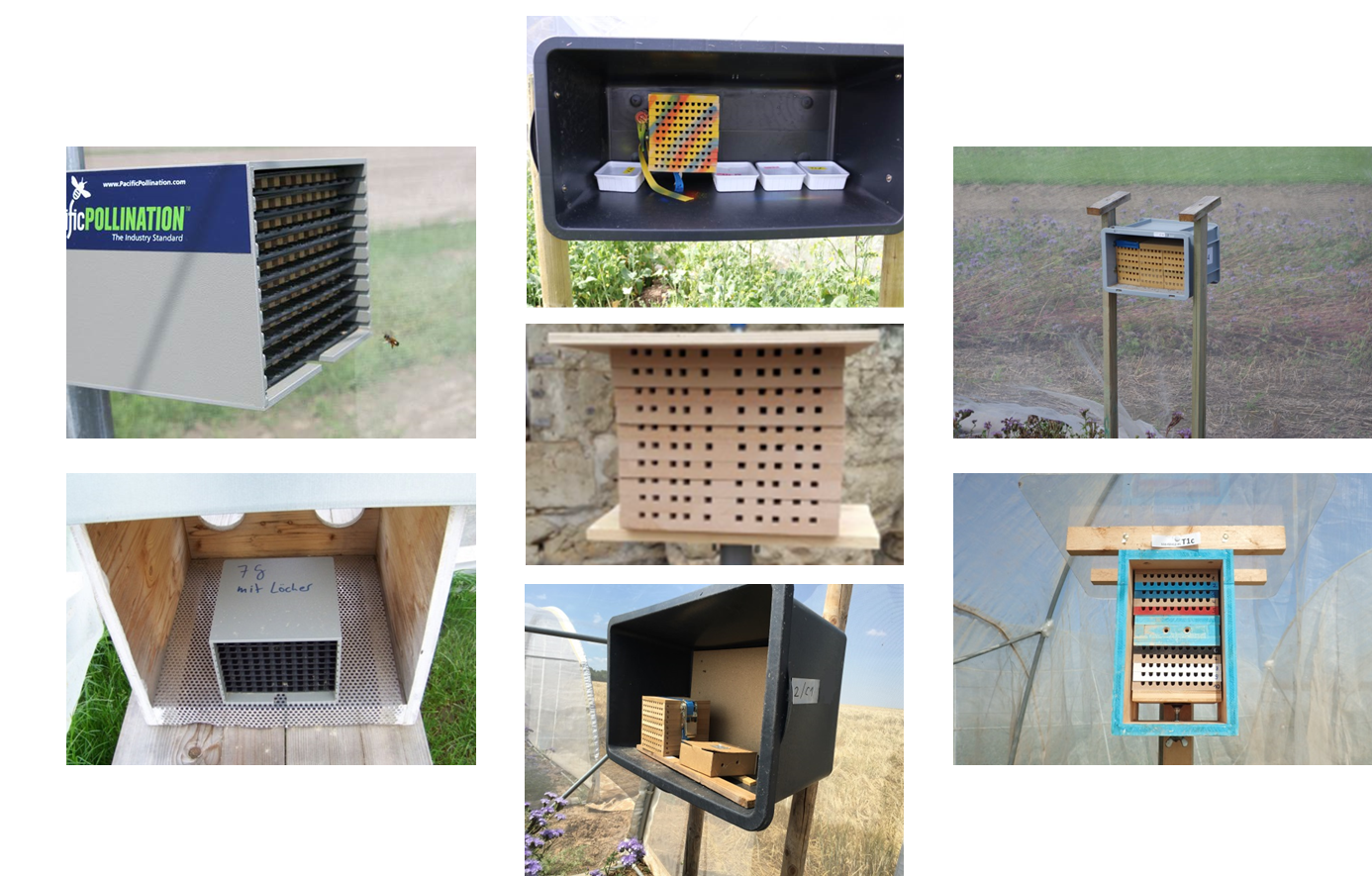


Figure SI-1. Pictures of different types of nesting units with rainproof roofs or shelters used in the semi-field studies with *Osmia bicornis* and *Osmia cornuta*. Left: Plastic trays; Middle and right: Medium density-fiberboard (MDF) trays. The use of MDF trays is recommended as establishment of female bees at nesting units containing MDF trays was found to be significantly higher compared to plastic trays.


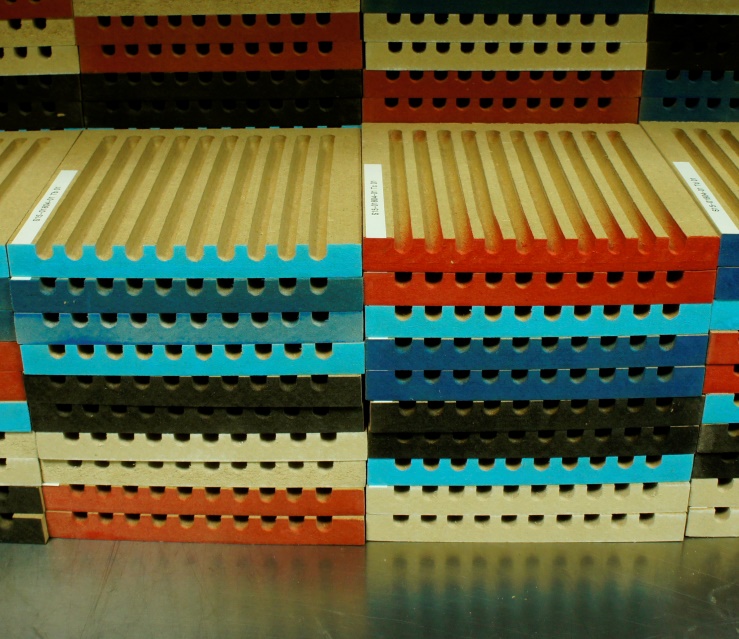

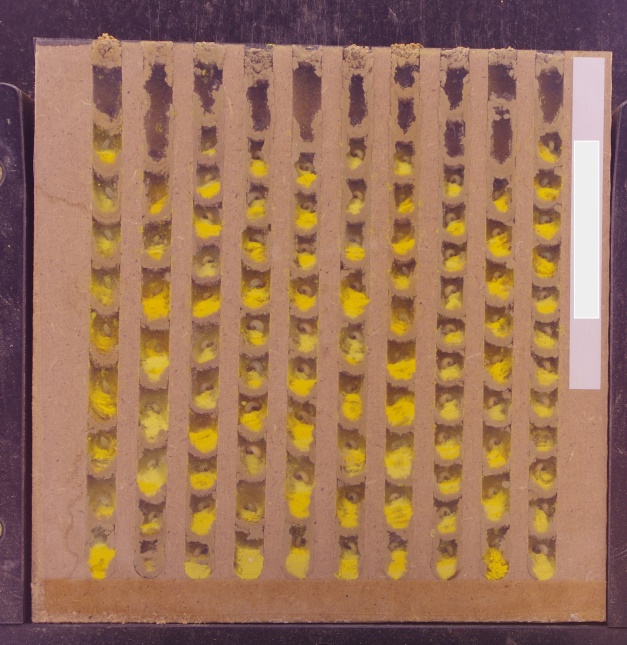


Figure SI-2. Picture of coloured trays with cavities (left) and a tray covered with a transparent plastic sheet to protect the eggs and young larvae and allow the marking of cells (right), which were used in the semi-field studies with *Osmia bicornis* and *Osmia cornuta*.

Table SI-2. Proposal on classes of minimal detectable differences (MDD) according to the EFSA Guidance Document for aquatic organisms (2013)

| Class |  | MDD | Comment |
| --- | --- | --- | --- |
| 0 |  | > 100% | No effects can be determined |
| I |  | 90-100% | Only large effects can be determined |
| II |  | 70-90% | Large to medium effects can be determined |
| III |  | 50-70% | Medium effects can be determined |
| IV |  | < 50% | Small effects can be determined |
